# Scientometric Analysis of Global Scientific Literature on Aging in Place

**DOI:** 10.3390/ijerph182312468

**Published:** 2021-11-26

**Authors:** Olugbenga Oladinrin, Kasun Gomis, Wadu Mesthrige Jayantha, Lovelin Obi, Muhammad Qasim Rana

**Affiliations:** 1 School of Architecture and the Built Environment, Faculty of Science and Engineering, University of Wolverhampton, Wolverhampton WV1 1LY, UK; O.Oladinrin@wlv.ac.uk (O.O.); L.Obi@wlv.ac.uk (L.O.); m.q.rana@wlv.ac.uk (M.Q.R.); 2 School of Property, Construction and Project Management, College of Design and Social Context, RMIT University, Melbourne VIC 3000, Australia; wadu.jayantha@rmit.edu.au

**Keywords:** aging in place, smart-home technologies, Gerontologist, VOSviewer

## Abstract

The amount of literature reporting “aging-in-place” studies has increased sharply in recent decades. However, the studies have taken a global view of the range and scope of the research that has taken place. This study presents a bibliometric analysis of the current status of the aging in place research themes published as scientific articles between 1970 and 2021, using the Web of Science database. VOSviewer software was employed to map and visualize the 1331 items of bibliographic data retrieved. The findings reveal a continuous growing trend in the publication of aging in place research. Most productive institutions derive from the USA. The International Journal of Environmental Research and Public Health is the most preferred Journal. The most popular research hotspots or areas include; older adults, aging, housing, dementia, long-term care, and technology, and their associations with the field of “aging in place” field were elucidated. This study offers several valuable insights to scholars, research institutions, and policymakers, enabling a better understanding of the developments in the aging in place research domain.

## 1. Introduction

There is evidence of a rapid increase in population aging across the globe [1,2]. Countries across the world are confronted with significant challenges of an aging population. For instance, the USA [3], Great Britain [4], China [5], India [6], Japan, which was tagged a super-aging society [7], Australia [8], Hong Kong [9], and some regions in Europe [10] non-exclusively, are few examples of nations facing the challenges associated with population aging. According to United Nations estimates, it is projected that the global population over the age of 60 years will reach 1 billion by 2020 and almost 2 billion by 2050 [11]. Even though the numbers did not reach the anticipated projection in 2020, the aging population over 65 years remains critical at 727 million persons [12]. More so, it has been estimated that the annual net increase of those aged 65 years or older will continue to exceed 10 million people over the next decade [4]. Accordingly, 26 countries had over 2 million older people in 1990, but this extended to 31 nations by the year 2000 [13]. The United Nations has also projected that the number of people aged 80 or older is estimated to more than triple between 2017 and 2050, increasing from 137 million to 425 million globally [14]. All these projections imply increased demand for extra health care as older people experience more chronic conditions [15], aging-friendly homes [16], as well as pensions for older populations [17]. Population aging, therefore, is a significant concern for key stakeholders, governments, policymakers, and researchers across the globe.

This rapid increase in population aging necessitates a reevaluation of conventional economic, political, and social policies to mitigate the potential problems associated with population aging [18,19]. It has been established that scientific advance is central to the economic and cultural development of a country [20]. Hence, an objective evaluation of the quality of the scientific publications of researchers and research organizations is vital to recognize a country’s present position and its potential for development in given domains [21,22]. As a component of science policy, it is necessary to track emerging research developments such as new fields and hotspots (i.e., areas of research concentration) worth special funding efforts or areas of growth and decline [23]. One of the approaches to forestalling the many challenges presented follows the recognition of the “aging in place” principle. Over the past few decades, concerned policymakers and elderly caregivers have advocated the conceptualization of aging in place as a realistic and valuable goal [24]. Golant [25] explores the current role of family support, housing, and care services in the private sector, government programmes, along with the promise of smart-home technology, creative planning, and long-term care strategies to ensure elderlies not only age in place but also age successfully in the right place. Consequently, aging in place has become an important area of interest in environmental gerontology and is the strong wish of both the elderly and their caregivers [24,26]. It is evident that not all older adults wish to age in place, especially those living in unsuitable or unsafe housing [27]. However, a recent study revealed that population aging already has, and will continue to have, significant consequences and effects in all areas of life; in the economic area, it is predicted that population aging would impact economic growth, employment, trade, labour markets, taxes and wealth and property transition from one generation to the next [28]. The concept of aging in place means that older people can age in a comfortable place and live in a familiar community with an emotional attachment to a long-term home. The term “place” refers to the home, community, or any physical space that people occupy and find significant. The larger part of older individuals needs to age-in-place, stay as independent, dynamic, and autonomous as long as possible, and live at domestic encompassed by family and companions [29,30]. The majority of older people aged 65 years and older are happier and more physically stable than earlier generations, indicating that older adults will live independently in the community later in the future [31]. Meanwhile, the aging of populations is expected to result in increased demand worldwide for long-term home care services [32]. Home-based care programs and age-friendly communities are some initiatives that reduce disability and promote aging in place [33,34]. However, aging in place operates in so many interacting ways that it requires proper policy and research attention [35].

Thus, aging in place has received, and is receiving, great attention from policymakers and scholars, with a surge in quantity and substance in the literature [24]. This surge may present a risk because of the difficulties in comprehending the existing state of the body of knowledge and the possibility of disregarding important questions and areas for research and practice improvement [36]. Avoiding this scientific risk requires a rigorous analysis of the domain. Up till now, this has not been sufficiently addressed. Past review studies on aging in place [24,37,38] (Graybill, McMeekin, & Wildman, 2014; Peek et al., 2014; Vasunilashorn, Steinman, Liebig, & Pynoos, 2012) have adopted a qualitative and narrative synthesis approach, based upon manual appraisals and by its nature subjective. Subjectivity is primarily conceptualized as how the investigation is affected by the researcher’s viewpoints, values, social encounters, and perspective. Hence, using such a qualitative approach as a validity test remains subjective. The scientometric review is quantitative, objective, and more reliable [39]. Markoulli, Lee, Byington, and Felps [40] posited that qualitative, manual reviews could not be applied to a comprehensive overview of an intellectual structure. More so, as global research on aging in place deals with several diverse views and issues, most bibliographic studies have exclusively focused on specific and limited aspects of aging in place. For example, while some review studies [38,41] focused on using technology to assist aging in place, another study by Graybill et al. [37] focused on the cost-effectiveness of aging in place. Rowlands [42] posits that bibliometric analysis offers greater transparency and the prospect of innovation in an environment that has really become a little jaded.

As aging is a prevalent phenomenon, the question is, how has the development of the research literature on this topic fared over the year? More precisely, the following research questions are addressed: (1) Who are the geographic contributors to aging in place research, and how have contributions evolved over the past years? (2) Which countries and organizations attract the most citation activity and by whom? (3) What form do international collaborations take based on publication co-authorship relationships? (4) How have research fronts changed over time based on the prevalence and co-occurrence of author keywords? (5) What are the related and relevant sources for the publication of aging in place research? Thus, this study aims to provide a systematic overview and identify future aging in place research trends. Because of the vast increase in literature on aging in place, bibliometric methods were employed to provide a quantitative analysis of the output (measured by the number of publications) and impact (measured by the number of citations). Although there are ongoing discussions about the reliability of specific bibliometric indices, the importance of evaluating the productivity of scientific research through the analysis of the quality of the publication and the corresponding citation data cannot be undermined [43]. However, the shortcoming of bibliometric analysis is the risk of presenting figures for the sake of statistics, with little comprehension of what they mean [42]. The analysis helps to answer the questions on the development and characteristics of the field of aging in place. Furthermore, analysis enables the identification of the most productive and influential articles, authors, core journals, countries, and organizations, together with information about the extent of cooperation among them. The analysis also enables identification of the extent of globalization existing within the research domain, leading topics, and potential gaps [44]. The bibliometric analysis offers informative guidelines for journal editors, policymakers, and researchers by providing information on research trends, those productive authors, active institutions, and research hotspots. When making funding decisions and promoting the growth of research opportunities and weaknesses, policymakers focused on such bibliometric knowledge and assessments [45].

## 2. Methodology

**Figure 1:**
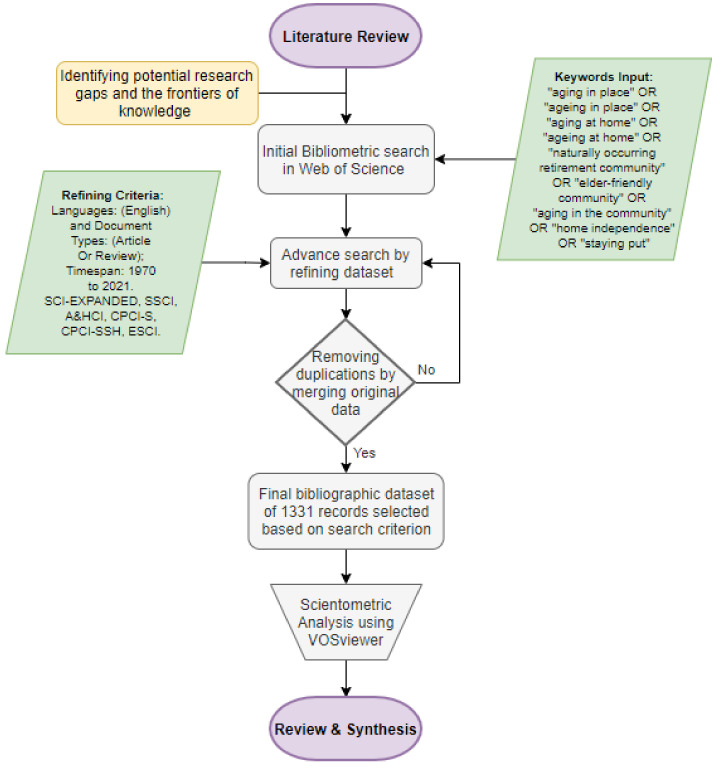
Methodology of the study.

The essence of the literature review study is to map and appraise the body of literature to identify potential research gaps and the frontiers of knowledge [46]. Structured literature reviews follow a systematic process including iterative cycles based on appropriate specified search keywords, followed by a bibliographic literature search, using an appropriate database, and a completing analysis [47]. Several researchers have used the bibliometric and scientometric review to evaluate the literature growth patterns, core journals, productive authors, influential institutions, contributing countries, research output performance, and research hotspots in a given field [48,49]. Bibliometric and scientometric analyses were employed in this study (see Figure 1). When conducting a literature review, Rowley and Slack [50] recommend a structured methodology for retrieving relevant resources, devising a mind map to organize the literature review, writing the review study, and developing the bibliography. A similar approach to bibliometric analysis by Sweileh et al. [51] was followed in this study.

### 2.1. Identification and Collection of Bibliographic Data

The Web of Science (Core Collection) was searched to collect bibliographic data used for the bibliometric analysis in this study because the Web of Science core collection contains comprehensive literature databases with high quality and influential articles [52]. Likewise, the Web of Science has been adjudged the most reliable scientific database [53], with the most reliable download function [54]. Moreover, the Web of Science core collection has advantages over other bibliographic databases such as Scopus. First, the citation matching algorithm in Scopus appears to need improvement when compared to Web of Science [55]. Second, duplicate articles in Scopus are a key source of data quality issues [56]. Hence, we chose to limit our search to the Web of Science only. A “topic” search was used based on search terms in the title, abstract and keywords, and keywords plus [57]. A wide range of terms representing the idea of aging in place identified by Vasunilashorn et al. [24] was used. The overall search string was as follows: TOPIC: (“aging in place” OR “ageing in place” OR “aging at home” OR “ageing at home” OR “naturally occurring retirement community” OR “elder-friendly community” OR “aging in the community” OR “home independence” OR “staying put”). Refined by: Languages: (English) and Document Types: (Article Or Review); Timespan: 1970 to 2021. SCI-EXPANDED, SSCI, A&HCI, CPCI-S, CPCI-SSH, ESCI. Due to the different language used between countries and cultures, as opined by Vasunilashorn et al. [24], the chosen search terms have restricted the inclusion of some publications by carefully removing unrelated research areas from the Web of Science before exporting the materials (e.g., agriculture and zoology). Only articles and reviews published in journals were involved as these are considered “certified knowledge” [58]. The knowledge contained in journals has already been subjected to a critical review and has succeeded in gaining approval from the research community; thus, enhancing the reliability of the analysis results. The search was conducted in September 2021, and 1331 records met the search criterion, becoming the bibliographic dataset. The data were downloaded as text files for analysis purposes. Web of Science searches are not sensitive to hyphenation [57]; thus, the search returned occurrences of duplications such as “ageing-in-place” and “aging in-place”; “older people” and “older adults”. All the duplications were merged in the original bibliographic data files before the analysis was concluded.

### 2.2. Method of Analysis

Traditionally, bibliometric analyses have been categorized into two types; whether the analyses yield activity or relationship indicators [58]. Activity yielding indicators present data conveying the force of impact or strength of the influence of research efforts, while relationship indicators trace the links and interactions between different items, such as researchers, documents, and keywords. VOSviewer software (version 1.6.17) (Lens, Brisbane, Austrilia) was used to obtain these indicators using bibliographic data to build a network of co-authorship, co-occurrence, and co-citation analyses. VOSviewer was used to combine both activity yielding and relationship indicators analyses. The software was used to create knowledge maps of the identified productive authors, core journals, contributing countries and organizations, influential documents, and co-occurring keywords. VOSviewer is a freely available software program developed for constructing and viewing bibliometric maps. Unlike most computer programs (such as VantagePoint and CiteSpace) used for bibliometric mapping, VOSviewer is highly responsive to the graphical representation of bibliometric maps and useful for presenting large, easy-to-interpret, bibliometric maps [59]. VOSviewer has been used in analysing scientific outputs in different research fields, such as tourism and sustainability [60], ground-penetrating radar [61], and communication [62].

## 3. Results and Discussion

**Figure 2:**
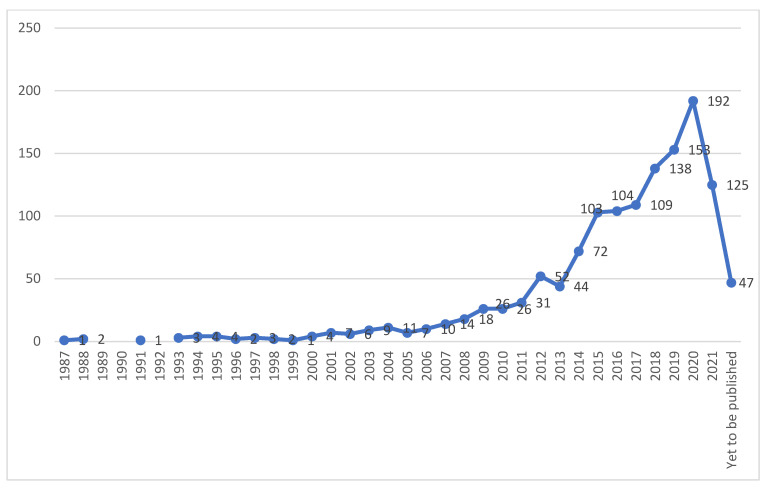
Number of publications from 1987 to 2021 in the Web of Science.

The 1331 published research articles were analysed, and the results were presented. Figure 2 reveals increased research in the aging in place domain. The figure shows an evident rise in the number of articles published on aging in place between 2010 and 2020, with the years 2019 (153 articles) and 2020 (192 articles) accounting for the most articles. Reasons for increased publications since 2010 was highlighted as a preference on maintaining the independence of older persons, emphasis of technology on non-institutional care, availability of grants in fostering aging in place, cost escalation of long-term institutional care, in addition to contemporary reforms and policies implemented [24,63]. The rapid decrease in publications for the years 2021 and 2022 is due to the incomplete bibliographic data records. This trend will probably continue to increase in the future research carried out. Hence, further analysis is required to gain more insights into the research direction in this domain.

### 3.1. Co-Authorship Analysis

The “co-authorship” identification is one of the main options provided by the Create Map wizard in VOSviewer. Co-authorship network analysis includes reliable algorithms that can track almost every aspect of scientific collaboration [64]. Hosseini et al. [36] described co-authorship as a shorthand for scientific collaboration. Co-authorship network analysis helps evaluate the collaborative behaviour of researchers, organizations, and countries in novel ways by disclosing the collaborative structure and information about the centrality of network participants. The wide range of applications indicates the adaptability of the information retrieved using this technology and offers new avenues for research collaboration. It enables one to comprehend the research structure on specific issues, the growth of research networks through time, and the participation of a certain institution or nation in a specific network [65]. Given this, co-authorship analysis was used in this study to create maps of authors, organizations, and countries.

#### 3.1.1. Authors

A total number of 3901 researchers participated in the 1331 bibliographic documents. However, in VOSviewer, the minimum number of documents for any author was set at five publications for clarity, which produced 40 authors meeting the threshold. This is to avoid the overlapping of many authors with fewer publications in the subsequent analysis of network visualization. The threshold was decided after several iterations, with five documents producing sufficient clarity. Moreover, articles with multiple authors were counted in full rather than proportionately to avoid confusion in their link strength. For each of the 40 authors, the corresponding number of citations and the total strength of their citation links with other authors were calculated as shown in Table 1. The total link strength attribute is used to evaluate the total strength of the co-authorship links of a given researcher with other researchers [66]. Szanton S.L. of the Johns Hopkins University in the United States produced the highest number of publications (18), joint with other authors and the highest total link strength (20). Thus, Rantz M.J. is the most influential author in aging in the place research domain. Rantz M.J. follows this with 15 submissions (link strength = 35) and Greenfield E.A. with 14 submissions (link strength = 13). Although the identity of this author showed as two different names, Rantz M.J. (nine articles) and Rantz M. (six articles), it was found that the two names were referring to the same author. All the influential authors are from the United States, indicating the extent of the research effort made those on aging in place. The findings seem like scholars in the United States have been at the forefront of concern about the global demographic shift, facilitating and championing the transition to ensure older people live comfortably in the places they desire. This could indicate that the US is a large country with an active aging society and high rates of funding for research.

**Figure 3:**
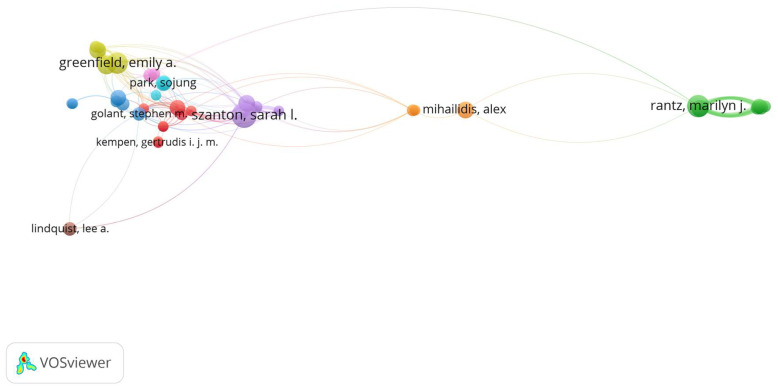
Network visualization of highly productive authors.

With such knowledge of the contemporary scientific collaboration networks, access to specialities, funds, expertise, and research productivity can be enhanced in this research domain [36]. Such knowledge is also crucial to broadening academic collaboration and communication by reducing isolation in research via the tracking of and connecting with investigators in various regions. Authors with minimum productivity of five documents were “visualized” using the VOSviewer technique. Figure 3 is a network visualization of highly productive authors based on the number of their publications. The map shows 40 circles, each representing one researcher with close circles indicating research collaborations between authors. These circles are clustered into ten, representing ten research communities. The lines in the map represent a link defining a connection between two scholars, indicating the number of co-authored publications. Through this collaborative practice, researchers build learning networks, promote different ways of thinking, and inspire solutions to research problems.

#### 3.1.2. Organizations/Institutions

Many institutions from all over the world publish aging-in-place-related research papers. Table 2 presents the top ten institutions with the highest number of publications to identify the most productive ones. Out of 1304 organizations identified from the bibliographic data, only 121 meet the threshold of five publications. Table 2 shows the top-performing research institutions, their geographic locations, and the number of publications they contributed to aging-in-place research. The most active institutions in the field were in the USA. This corroborates the previous findings on productive authors in that the authors were from institutions in the US. The University of Missouri ranks first in terms of published articles related to aging in place, with 32 documents, followed by the University of Maryland and the University of Toronto with 25 publications each.

**Figure 4:**
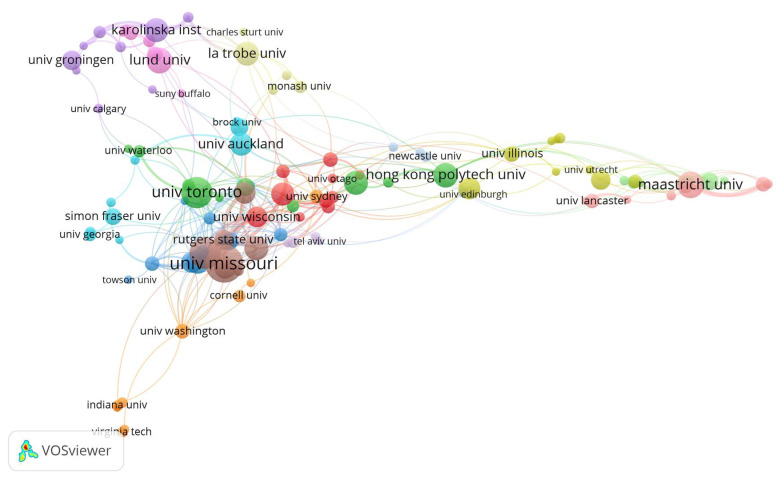
Network visualization of contributing organizations.

A network visualization map showing collaboration among those research institutions that have produced a productivity minimum of five documents is shown in Figure 4. The thickness of the lines connecting any two institutions indicates the strength of collaboration. Figure 4 demonstrates the operational closeness of the institutions in terms of collaboration and their ranking to serve as research centres. In performing aging-in-place studies, organizations from the United States, the United Kingdom, Europe, and Asia have succeeded in establishing collaborative relationships with each other.

#### 3.1.3. Countries

Fifty-five countries contributed to the publication of the retrieved documents, but only 30 met the threshold of five publications. The 10 most prolific countries are listed in Table 3. On the map of countries, the USA had the most significant number of publications (524), followed by Canada (139), the UK (127), Australia (110), and the Netherlands (92). Based on the analysis of English language publications, the findings show that the USA has moved further and faster in the aging in place research field than any other major research funding country. The result is not surprising because the USA has been leading the world in significant publication output. The finding also revealed that the significant contributions to research on aging in place derive from developed nations, whereas the research outputs from less developed nations are comparably low. This study does not identify the regional focus of the research carried out as it was beyond the scope of this study. The data does not identify that the research output of the developing countries is low compared to the developed countries, e.g., authors in developed countries could carry out research on developing countries (and vice versa). Nonetheless, the study identified an eminent lack of research from authors located in developing countries. Further reasons for this need to be researched with specific emphasis on regional focus.

**Figure 5:**
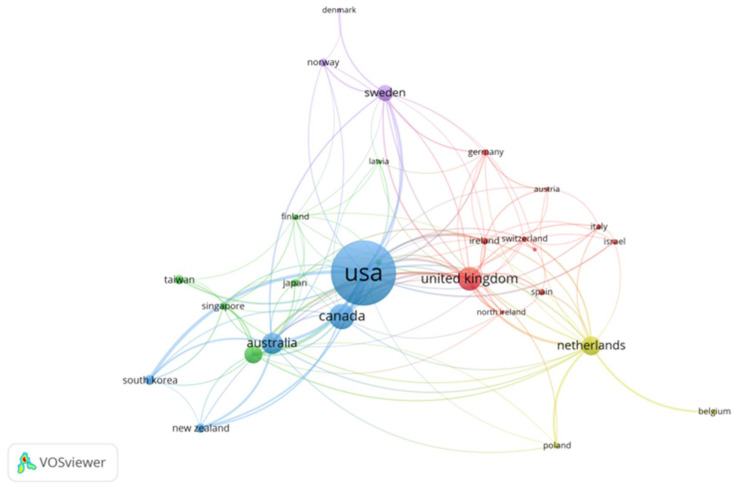
Network visualization of countries.

Figure 5 illustrates the degree of collaboration among countries with a threshold of five documents each. The network includes 32 countries distributed over six different clusters, each country with a different colour. There are 134 links, which is an indication of good networking. The thickness of the link between any two countries indicates the strength of collaboration. The most substantial collaboration was between the following pairs of countries: USA–China (link strength = 15); USA–Canada and USA–South Korea (link strength = 14); England–Scotland (link strength = 12); USA–Australia (link strength = 11); USA–Sweden (link strength = 10). Hence, the high level of commitment of the country to aging in place has resulted in significant collaboration from other countries.

### 3.2. Co-Occurrence

Occurrences attributed in VOSviewer indicate the number of documents in which a specified keyword occurs [66]. Co-occurrence networks are graphs that show how frequently variables appear together. They are extensively used in text mining, where co-occurrence counts how frequently two words appear together at a sample site or how frequently two terms appear in a single document. A co-occurrence network allows us to investigate several pairs of co-occurring variables at the same time. Each variable is represented by a node or point in the construction of a co-occurrence network. The co-occurrence of two variables is represented by an edge, or connection, linking two nodes. Primary research focuses can be discovered by analysing the keywords found within the articles [52]. A keywords network provides a sound picture of a knowledge domain, enabling understanding of the topics covered and the interrelationship between various topics [67].

#### Keywords

**Figure 6:**
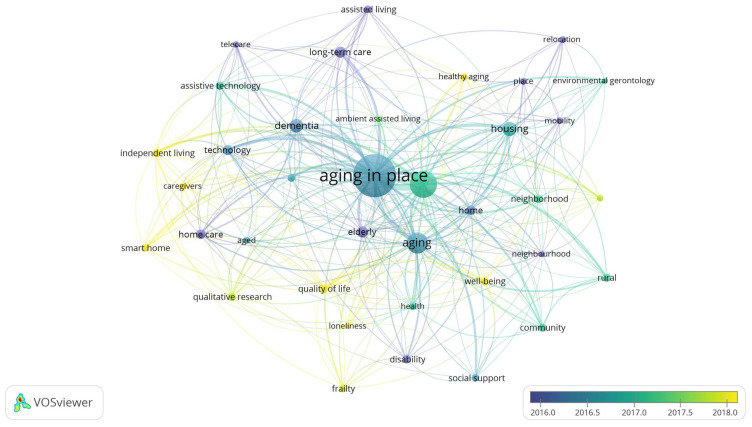
Overlay visualization of keywords.

VOSviewer technique was used to map the keywords, using author keywords rather than all keywords to achieve a reproducible and readable map [51,68]. With a threshold of 15 minimum occurrences, 37 keywords met the threshold out of a pool of 3045 keywords drawn from 1331 papers. After five attempted iterations, a minimum threshold of 15 produced a clear network visualization and was used for the analysis. The most popular keywords or research hotspots include: older adults, aging, housing, dementia, long-term care, and technology, in accordance with “aging in place”. These keywords depict the main areas of current aging in place research. Obviously, the most popular keywords (i.e., aging in place) in Figure 6 occur because of their inclusion in the search keywords chosen for this study. The strength of the link connecting two keywords reflects the number of articles in which the keywords appear together, revealing the association of their respective research focuses [69].

The strongest links are among the following pairs of keywords: aging in place–older adults (link strength = 88); aging in place and aging (link strength = 30); aging in place–housing (link strength = 27); aging in place–dementia (link strength = 21); aging in place–technology (link strength = 18); aging in place–independent living (link strength = 16). Therefore, older adults, aging, housing, dementia, technology, and independent living are the research hotspots on aging in place studies. This reveals multiple issues surrounding aging in place among diverse populations, thus creating various research directions for scholars in this field. Vasunilashorn et al. [24] put it that “third, aging in place is not a one-size-fits-all concept”. VOSviewer commonly lists together keywords with the same colour. Overlay visualization was used to group the keywords according to their average year of occurrence, using VOSviewer selected years (2016–2018). The closer the colour to purple, the earlier the occurrence of the keywords, and the closer the colour to yellow, the more current or recent the keywords. It can be deduced that current research focuses on smart-homes, independent living, social care, frailty, quality of life, and healthy aging, as they all appear in yellow.

Older people will continue to live in their familiar surroundings for as long as they are able. To increase the age-friendliness of communities, housing activists and older citizens can address individual and community-wide challenges such as loneliness, dementia, long-term care, and disability. The growing population of older persons and their desire to age in place pose considerable healthcare and housing issues [70]. Previous studies on elderly housing primarily focused on senior housing, sheltered housing, nursing homes, and community dwellings. The global ageing issue emphasizes the gap between traditional housing and the fundamental level of housing necessary to allow individuals to dwell in their houses as their requirements evolve. Several studies have found that housing and neighbourhood surroundings influence the psychological well-being of the elderly [71]. Incorporating visitability and universal design elements into home development can increase the inventory of accessible houses available to older individuals and facilitate ageing in place [72]. The “neighbourhood” is another important factor in aging in place. As many older persons express a desire to age in place, it is critical to understand how neighbourhood change might help or hinder their capacity to do so [73]. As smart home automation technology advances, there is rising interest in its potential to enable older persons to age in place [74]. While the usage of smart technology in residential settings is increasing, research on how such technologies might give chances for safely and productively ageing in place by incorporating physical exercise into everyday routines and lowering sedentariness is limited [70].

### 3.3. Co-Citation Analysis

Co-citation analysis entails tracking pairs of publications that are referenced together in the source articles. The data gathered in the co-citation study were counts of the number of times two journal titles were jointly cited in later works. It is considered that the more two journals are referenced together, the more closely they are related. The co-citation analysis of sources enables the identification of the most cited and highly influential research documents and author journals responsible for aging in place studies. Academic journals play an important role in disseminating research findings [75]. Thus, it is critical to investigate the significant research outlets in the region when analysing the research trend. The goal is not to promote journals but to inform researchers about the best outlets and platforms for disseminating their research findings to have maximum impact in academia and industry.

#### Journals

Sources are referred to as journals in the VOSviewer platform. From the data analysis, it has been found that all the bibliographic references obtained from the Web of Science were included in 473 journals. Of these, only 24 meet the threshold of 10 publications. The minimum threshold was decided after five attempted iterations, with 10 producing a clear network visualization. As shown in Table 4, The International Journal of Environmental Research and Public Health is the topmost Journal with 45 articles, cited 372 times, with the highest total link strength of 82 (the link strength between two nodes denotes the frequency of co-occurrence of the journals being represented by the nodes), followed by Gerontologist, which published 43 articles, with a total link strength of 1956. Aging and Society (40 articles) and Journal of applied gerontology (32 articles) occupied the third and fourth positions, respectively. These journals have received the highest number of citations and total link strengths. These journals, therefore, have made significant contributions to aging in place studies. These findings help identify the core sets of journals, which publish the most in the field of aging in place. Researchers, practitioners, and librarians are informed of the journals they might prioritize in retrieving relevant sources, in publishing findings, and for inclusion in a library collection.

**Figure 7:**
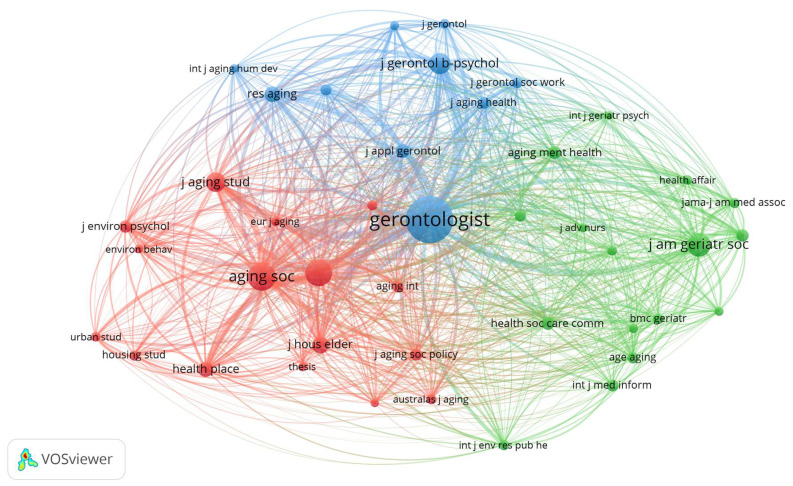
Network visualization of journal co-citations.

Figure 7 shows a network visualization map of co-citation analysis for journals with minimum citations of 150. With the largest circle size, Gerontologist received the highest number of citations (1992), with the highest number of links with other journals (total link strength = 27,884), indicating that this journal was co-cited within most other journals. Journals in the same cluster with the same colour are commonly co-cited. In essence, Gerontologist has the highest number of co-cited articles in aging in place related studies, and it also belongs to the broadest network.

## 4. Conclusions

This study aimed at presenting a bibliometric analysis of the current status of the aging in place research themes. A global view of the publications produced in the research field of aging in place between 1970 and 2021 is presented in this paper. A total of 1331 original and review articles, published in 473 different peer-reviewed journals by a total of 3901 authors, were identified. The study focused on the Web of Science since it was deemed the most reliable and influential database for bibliometric research. The keywords selected for the bibliometric study was identified by the prominent research themes under aging in place.

The concept of aging in place was not common during the 1970s and 1980s, hence, the low number of publications. However, there has been a significant increase in publications from the 1990s upwards, with the most significant number of publications in recent years, in line with Vasunilashorn et al. [24]. The United States was the most productive in terms of the number of articles published in the English language. The International Journal of Environmental Research and Public Health is the journal that has published more articles in this area. Based on co-occurrence analysis, the research hotspots identified during the study include: older adults, aging, housing, dementia, long-term care, and technology, and their association with aging in place field. Moreover, the latest hotspots were identified, which may signify future research directions. A wide range of indicators was used in the study, including co-authorship, co-citation, and co-occurrence analyses, presented informatively from different perspectives so that interested readers can apply the results according to their interests and priorities. However, the findings should be cautiously interpreted to avoid misunderstanding in guiding future research.

The study can be viewed as the first step towards an objective analysis of the literature existing in the aging in place research field. The study identified housing, dementia, long-term care, and technology as emerging research focuses within the subject area. Noticeable contributions from the authors listed were prominent in underpinning future research focuses in the subject area. As bibliometric analysis is not static, for instance, concepts may gain or lose attention over time as more publications are released, relationships between authors, documents, and countries can be altered, and new research directions may emerge. Therefore, similar studies should be performed in the future to keep tracks of changes in the field. Nonetheless, the data analysed offer useful insights for guiding interested researchers and prioritizing future research efforts in aging in place studies. The analysis offers several insights that may aid aging in place researchers, educational institutions, and policymakers in their perception of the development of the field. Thus, this study has successfully achieved the primary objectives in recognizing the current context and future research trends in improving aging in place subject area.

The study further identified the degree of international collaborations and identified that most of the collaborations were carried out with the USA. Very limited collaborations were identified within Asian and Asian-pacific regions with the European regions. However, the study does not underpin the research carried out from developed and developing regions as the regional focus was not the scope of this study. Further research might be beneficial if more collaborations were carried out to identify the research emphasis from regional focus and weigh in the prominence of collaboration between Asian/Asian-pacific and European regions. Future research may also make data sources, such as Scopus, because Web of Science is not all-encompassing, and some critical articles might have been omitted. More so, the findings should be interpreted in line with the definition of aging in place given in this study. It is difficult to relate if all the included papers have anything to do with aging in place—some papers might be using the term as a buzzword in the title/abstract, while the inclusion of some papers published in languages other than the English language could alter the results.

## Figures and Tables

**Table 1:** The top 10 most productive authors.

Author	Institution	Country	Total Publications	Citations	Total Link Strength
Szanton, S.I.	Johns Hopkins University	USA	18	405	20
Rantz, M.J.	University of Missouri-Columbia	USA	15	428	35
Greenfield, E.A.	State University of New Jersey	USA	14	243	13
Lehning, A.J.	University of Maryland	USA	12	233	14
Skubic, M.	University of Missouri-Columbia	USA	10	338	24
Gitlin, L.N.	Drexel University, Philadelphia	USA	10	308	18
Scharlach, A.E.	University of California	USA	10	161	16
Mihailidis, A.	University of Toronto	Canada	10	169	0
Iwarsson, S.	Lund University	Sweden	9	183	10
Park, S.	Washington University	USA	9	64	7

**Table 2:** The top 11 most productive institutions.

Institution	Location	Number of Publication	Citations
University of Missouri	USA	32	688
University of Toronto	Canada	25	573
University of Maryland	USA	25	509
Maastricht University	Netherland	21	251
Lund University	Sweden	20	352
Hong Kong Polytechnic University	Hong Kong	19	142
Washington University	USA	18	219
University of Michigan	USA	18	475
Karolinska Institute	Sweden	18	171
La Trobe University	Australia	18	235
University of Florida	USA	18	221

**Table 3:** The top 10 most participating countries.

Location	Number of Publication	Citations	Total Link Strength
USA	524	8508	117
Canada	139	2714	47
UK	127	2223	61
Australia	110	1306	56
The Netherlands	92	1423	53
China	85	687	57
Sweden	73	779	41
South Korea	37	159	27
New Zealand	34	1034	21
Taiwan	34	381	10

**Table 4:** The top 10 most productive journals.

Source Journal	Documents	Citations	Total Link Strength
International Journal of Environmental Research and Public Health	45	372	82
Gerontologist	43	1956	295
Ageing and Society	40	740	183
Journal of Applied Gerontology	32	313	78
Health and Social care in the community	30	289	45
Journal of Housing for the Elderly	29	145	86
BMC Geriatrics	27	298	42
Journal of Aging Studies	26	620	138
Research on Aging	17	392	69
Housing Studies	17	153	48

## Data Availability

Not Applicable.
